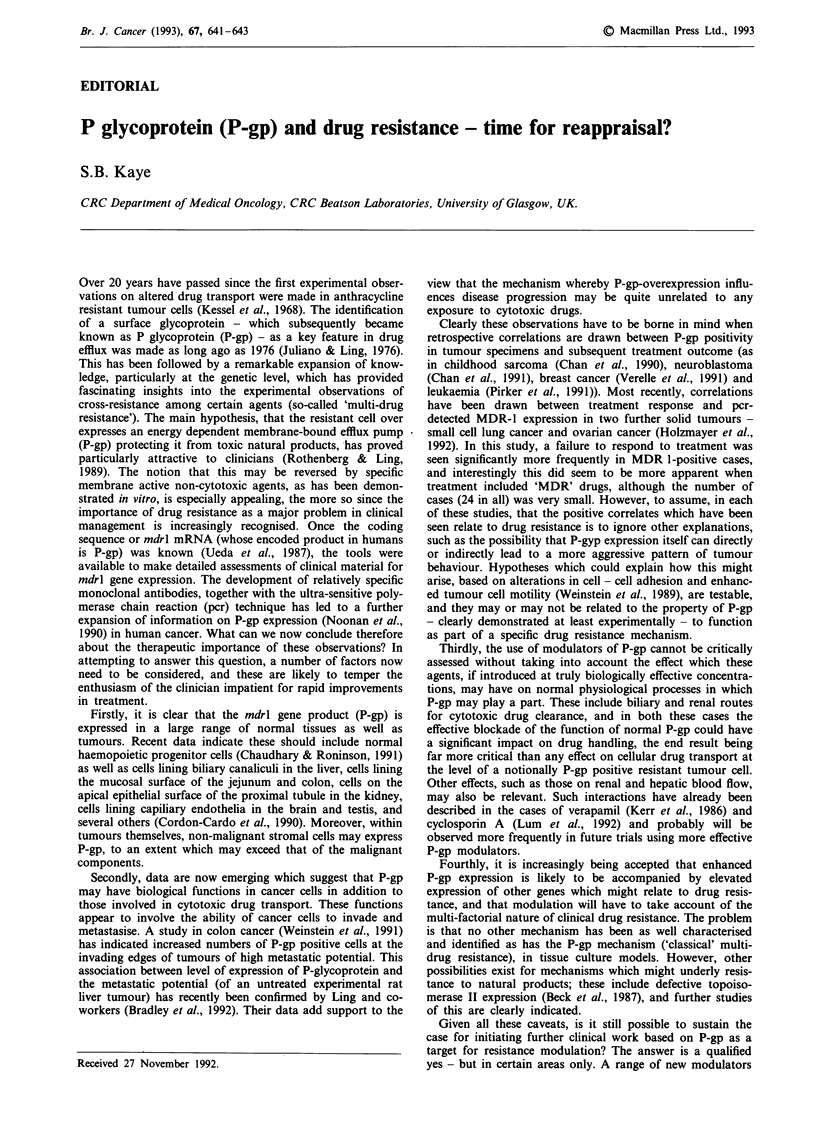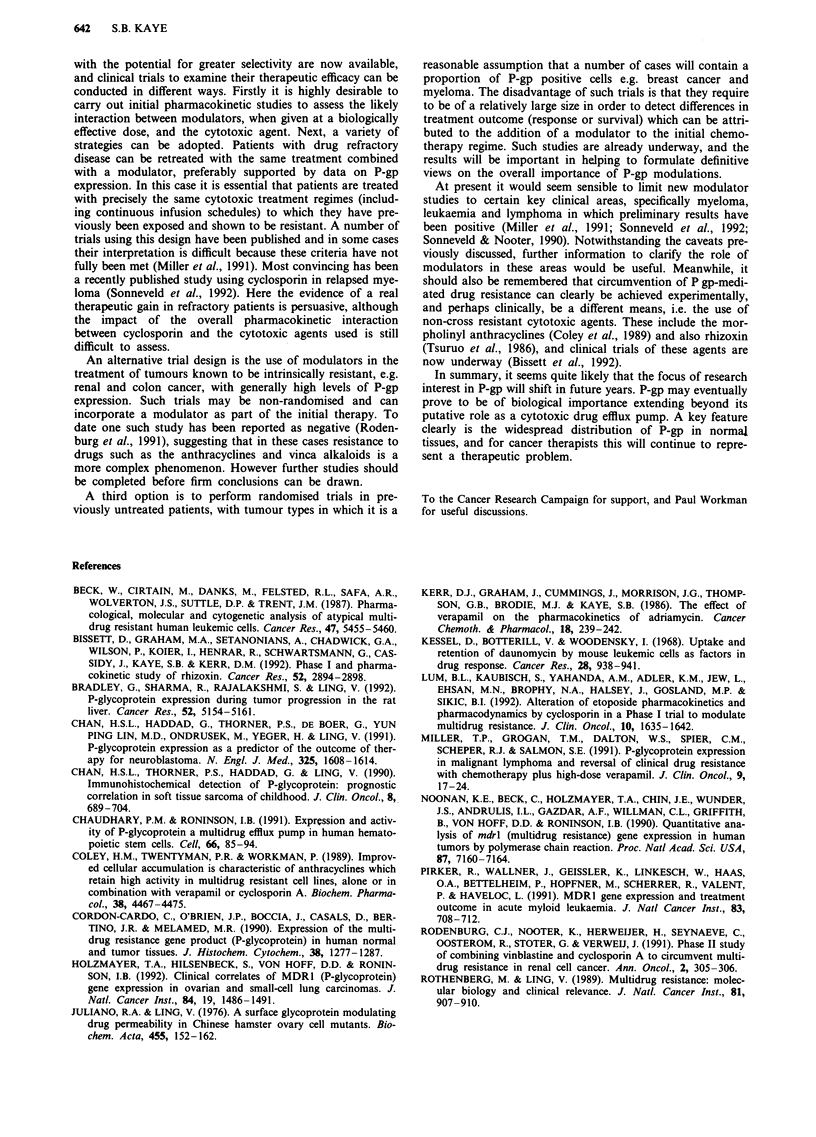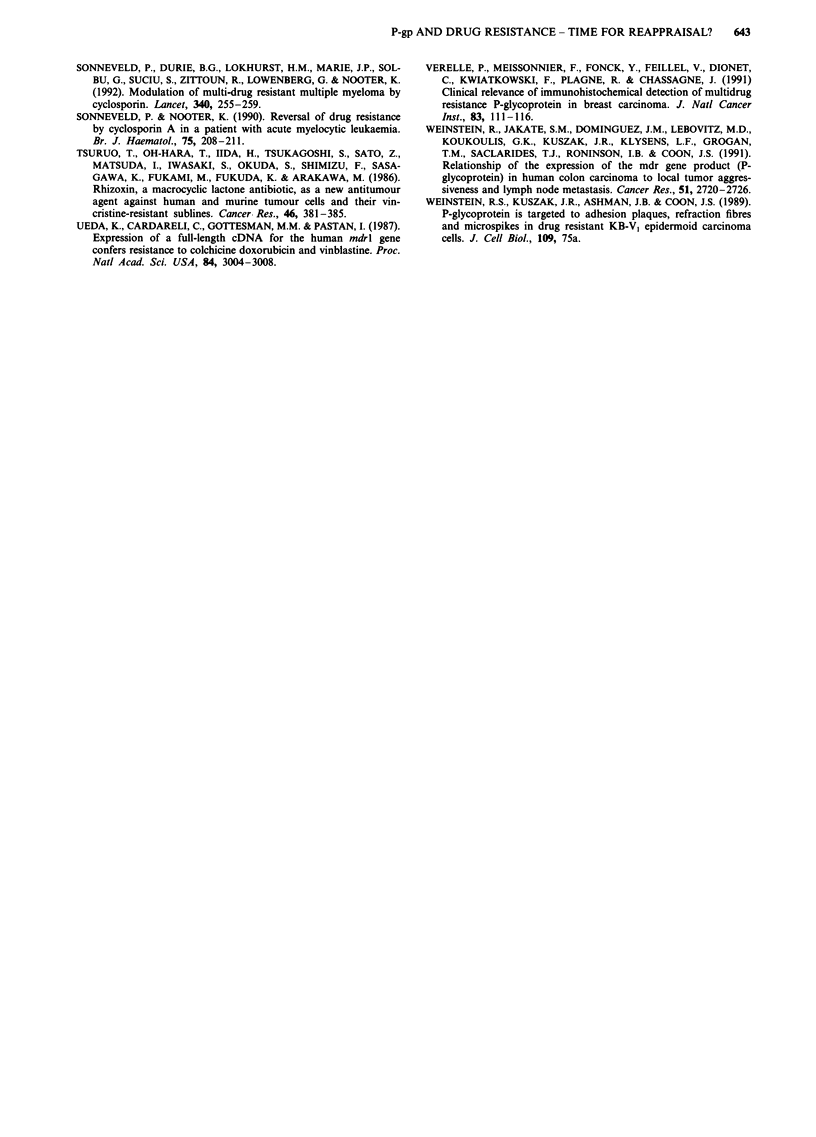# P glycoprotein (P-gp) and drug resistance--time for reappraisal?

**DOI:** 10.1038/bjc.1993.119

**Published:** 1993-04

**Authors:** S. B. Kaye


Br. J. Cancer (1993), 67, 641-643 © Macmillan Press Ltd., 1993

EDITORIAL

### P glycoprotein (P-gp) and drug resistance – time for reappraisal?

S.B. Kaye

*CRC Department of Medical Oncology, CRC Beatson Laboratories, University of Glasgow, UK.*

Over 20 years have passed since the first experimental observations on altered drug transport were made in anthracycline resistant tumour cells (Kessel *et al.*, 1968). The identification of a surface glycoprotein – which subsequently became known as P glycoprotein (P-gp) – as a key feature in drug efflux was made as long ago as 1976 (Juliano & Ling, 1976). This has been followed by a remarkable expansion of knowledge, particularly at the genetic level, which has provided fascinating insights into the experimental observations of cross-resistance among certain agents (so-called 'multi-drug resistance'). The main hypothesis, that the resistant cell over expresses an energy dependent membrane-bound efflux pump (P-gp) protecting it from toxic natural products, has proved particularly attractive to clinicians (Rothenberg & Ling, 1989). The notion that this may be reversed by specific membrane active non-cytotoxic agents, as has been demonstrated *in vitro*, is especially appealing, the more so since the importance of drug resistance as a major problem in clinical management is increasingly recognised. Once the coding sequence or *mdr*1 mRNA (whose encoded product in humans is P-gp) was known (Ueda *et al.*, 1987), the tools were available to make detailed assessments of clinical material for *mdr*1 gene expression. The development of relatively specific monoclonal antibodies, together with the ultra-sensitive polymerase chain reaction (pcr) technique has led to a further expansion of information on P-gp expression (Noonan *et al.*, 1990) in human cancer. What can we now conclude therefore about the therapeutic importance of these observations? In attempting to answer this question, a number of factors now need to be considered, and these are likely to temper the enthusiasm of the clinician impatient for rapid improvements in treatment.

Firstly, it is clear that the *mdr*1 gene product (P-gp) is expressed in a large range of normal tissues as well as tumours. Recent data indicate these should include normal haemopoietic progenitor cells (Chaudhary & Roninson, 1991) as well as cells lining biliary canaliculi in the liver, cells lining the mucosal surface of the jejunum and colon, cells on the apical epithelial surface of the proximal tubule in the kidney, cells lining capiliary endothelia in the brain and testis, and several others (Cordon-Cardo *et al.*, 1990). Moreover, within tumours themselves, non-malignant stromal cells may express P-gp, to an extent which may exceed that of the malignant components.

Secondly, data are now emerging which suggest that P-gp may have biological functions in cancer cells in addition to those involved in cytotoxic drug transport. These functions appear to involve the ability of cancer cells to invade and metastasise. A study in colon cancer (Weinstein *et al.*, 1991) has indicated increased numbers of P-gp positive cells at the invading edges of tumours of high metastatic potential. This association between level of expression of P-glycoprotein and the metastatic potential (of an untreated experimental rat liver tumour) has recently been confirmed by Ling and coworkers (Bradley *et al.*, 1992). Their data add support to the view that the mechanism whereby P-gp-overexpression influences disease progression may be quite unrelated to any exposure to cytotoxic drugs.

Clearly these observations have to be borne in mind when retrospective correlations are drawn between P-gp positivity in tumour specimens and subsequent treatment outcome (as in childhood sarcoma (Chan *et al.*, 1990), neuroblastoma (Chan *et al.*, 1991), breast cancer (Verelle *et al.*, 1991) and leukaemia (Pirker *et al.*, 1991)). Most recently, correlations have been drawn between treatment response and pcr-detected MDR-1 expression in two further solid tumours – small cell lung cancer and ovarian cancer (Holzmayer *et al.*, 1992). In this study, a failure to respond to treatment was seen significantly more frequently in MDR 1-positive cases, and interestingly this did seem to be more apparent when treatment included 'MDR' drugs, although the number of cases (24 in all) was very small. However, to assume, in each of these studies, that the positive correlates which have been seen relate to drug resistance is to ignore other explanations, such as the possibility that P-gyp expression itself can directly or indirectly lead to a more aggressive pattern of tumour behaviour. Hypotheses which could explain how this might arise, based on alterations in cell – cell adhesion and enhanced tumour cell motility (Weinstein *et al.*, 1989), are testable, and they may or may not be related to the property of P-gp – clearly demonstrated at least experimentally – to function as part of a specific drug resistance mechanism.

Thirdly, the use of modulators of P-gp cannot be critically assessed without taking into account the effect which these agents, if introduced at truly biologically effective concentrations, may have on normal physiological processes in which P-gp may play a part. These include biliary and renal routes for cytotoxic drug clearance, and in both these cases the effective blockade of the function of normal P-gp could have a significant impact on drug handling, the end result being far more critical than any effect on cellular drug transport at the level of a notionally P-gp positive resistant tumour cell. Other effects, such as those on renal and hepatic blood flow, may also be relevant. Such interactions have already been described in the cases of verapamil (Kerr *et al.*, 1986) and cyclosporin A (Lum *et al.*, 1992) and probably will be observed more frequently in future trials using more effective P-gp modulators.

Fourthly, it is increasingly being accepted that enhanced P-gp expression is likely to be accompanied by elevated expression of other genes which might relate to drug resistance, and that modulation will have to take account of the multi-factorial nature of clinical drug resistance. The problem is that no other mechanism has been as well characterised and identified as has the P-gp mechanism ('classical' multi-drug resistance), in tissue culture models. However, other possibilities exist for mechanisms which might underly resistance to natural products; these include defective topoisomerase II expression (Beck *et al.*, 1987), and further studies of this are clearly indicated.

Given all these caveats, is it still possible to sustain the case for initiating further clinical work based on P-gp as a target for resistance modulation? The answer is a qualified yes – but in certain areas only. A range of new modulators

Received 27 November 1992.

with the potential for greater selectivity are now available, and clinical trials to examine their therapeutic efficacy can be conducted in different ways. Firstly it is highly desirable to carry out initial pharmacokinetic studies to assess the likely interaction between modulators, when given at a biologically effective dose, and the cytotoxic agent. Next, a variety of strategies can be adopted. Patients with drug refractory disease can be retreated with the same treatment combined with a modulator, preferably supported by data on P-gp expression. In this case it is essential that patients are treated with precisely the same cytotoxic treatment regimes (including continuous infusion schedules) to which they have previously been exposed and shown to be resistant. A number of trials using this design have been published and in some cases their interpretation is difficult because these criteria have not fully been met (Miller *et al.*, 1991). Most convincing has been a recently published study using cyclosporin in relapsed myeloma (Sonneveld *et al.*, 1992). Here the evidence of a real therapeutic gain in refractory patients is persuasive, although the impact of the overall pharmacokinetic interaction between cyclosporin and the cytotoxic agents used is still difficult to assess.

An alternative trial design is the use of modulators in the treatment of tumours known to be intrinsically resistant, e.g. renal and colon cancer, with generally high levels of P-gp expression. Such trials may be non-randomised and can incorporate a modulator as part of the initial therapy. To date one such study has been reported as negative (Rodenburg *et al.*, 1991), suggesting that in these cases resistance to drugs such as the anthracyclines and vinca alkaloids is a more complex phenomenon. However further studies should be completed before firm conclusions can be drawn.

A third option is to perform randomised trials in previously untreated patients, with tumour types in which it is a reasonable assumption that a number of cases will contain a proportion of P-gp positive cells e.g. breast cancer and myeloma. The disadvantage of such trials is that they require to be of a relatively large size in order to detect differences in treatment outcome (response or survival) which can be attributed to the addition of a modulator to the initial chemotherapy regime. Such studies are already underway, and the results will be important in helping to formulate definitive views on the overall importance of P-gp modulations.

At present it would seem sensible to limit new modulator studies to certain key clinical areas, specifically myeloma, leukaemia and lymphoma in which preliminary results have been positive (Miller *et al.*, 1991; Sonneveld *et al.*, 1992; Sonneveld & Nooter, 1990). Notwithstanding the caveats previously discussed, further information to clarify the role of modulators in these areas would be useful. Meanwhile, it should also be remembered that circumvention of P gp-mediated drug resistance can clearly be achieved experimentally, and perhaps clinically, be a different means, i.e. the use of non-cross resistant cytotoxic agents. These include the morpholinyl anthracyclines (Coley *et al.*, 1989) and also rhizoxin (Tsuruo *et al.*, 1986), and clinical trials of these agents are now underway (Bissett *et al.*, 1992).

In summary, it seems quite likely that the focus of research interest in P-gp will shift in future years. P-gp may eventually prove to be of biological importance extending beyond its putative role as a cytotoxic drug efflux pump. A key feature clearly is the widespread distribution of P-gp in normal tissues, and for cancer therapists this will continue to represent a therapeutic problem.

To the Cancer Research Campaign for support, and Paul Workman for useful discussions.

### References

BECK, W., CIRTAIN, M., DANKS, M., FELSTED, R.L., SAFA, A.R., WOLVERTON, J.S., SUTTLE, D.P. & TRENT, J.M. (1987). Pharmacological, molecular and cytogenetic analysis of atypical multidrug resistant human leukemic cells. *Cancer Res.*, **47**, 5455–5460.

BISSETT, D., GRAHAM, M.A., SETANONIANS, A., CHADWICK, G.A., WILSON, P., KOIER, I., HENRAR, R., SCHWARTSMANN, G., CASSIDY, J., KAYE, S.B. & KERR, D.M. (1992). Phase I and pharmacokinetic study of rhizoxin. *Cancer Res.*, **52**, 2894–2898.

BRADLEY, G., SHARMA, R., RAJALAKSHMI, S. & LING, V. (1992). P-glycoprotein expression during tumor progression in the rat liver. *Cancer Res.*, **52**, 5154–5161.

CHAN, H.S.L., HADDAD, G., THORNER, P.S., DE BOER, G., YUN PING LIN, M.D., ONDRUSEK, M., YEGER, H. & LING, V. (1991). P-glycoprotein expression as a predictor of the outcome of therapy for neuroblastoma. *N. Engl. J. Med.*, **325**, 1608–1614.

CHAN, H.S.L., THORNER, P.S., HADDAD, G. & LING, V. (1990). Immunohistochemical detection of P-glycoprotein: prognostic correlation in soft tissue sarcoma of childhood. *J. Clin. Oncol.*, **8**, 689–704.

CHAUDHARY, P.M. & RONINSON, I.B. (1991). Expression and activity of P-glycoprotein a multidrug efflux pump in human hematopoietic stem cells. *Cell*, **66**, 85–94.

COLEY, H.M., TWENTYMAN, P.R. & WORKMAN, P. (1989). Improved cellular accumulation is characteristic of anthracyclines which retain high activity in multidrug resistant cell lines, alone or in combination with verapamil or cyclosporin A. *Biochem. Pharmacol.*, **38**, 4467–4475.

CORDON-CARDO, C., O'BRIEN, J.P., BOCCIA, J., CASALS, D., BERTINO, J.R. & MELAMED, M.R. (1990). Expression of the multidrug resistance gene product (P-glycoprotein) in human normal and tumor tissues. *J. Histochem. Cytochem.*, **38**, 1277–1287.

HOLZMAYER, T.A., HILSENBECK, S., VON HOFF, D.D. & RONINSON, I.B. (1992). Clinical correlates of MDR1 (P-glycoprotein) gene expression in ovarian and small-cell lung carcinomas. *J. Natl. Cancer Inst.*, **84**, 19, 1486–1491.

JULIANO, R.A. & LING, V. (1976). A surface glycoprotein modulating drug permeability in Chinese hamster ovary cell mutants. *Biochem. Acta*, **455**, 152–162.

KERR, D.J., GRAHAM, J., CUMMINGS, J., MORRISON, J.G., THOMPSON, G.B., BRODIE, M.J. & KAYE, S.B. (1986). The effect of verapamil on the pharmacokinetics of adriamycin. *Cancer Chemoth. & Pharmacol.*, **18**, 239–242.

KESSEL, D., BOTTERILL, V. & WOODENSKY, I. (1968). Uptake and retention of daunomycin by mouse leukemic cells as factors in drug response. *Cancer Res.*, **28**, 938–941.

LUM, B.L., KAUBISCH, S., YAHANDA, A.M., ADLER, K.M., JEW, L., EHSAN, M.N., BROPHY, N.A., HALSEY, J., GOSLAND, M.P. & SIKIC, B.I. (1992). Alteration of etoposide pharmacokinetics and pharmacodynamics by cyclosporin in a Phase I trial to modulate multidrug resistance. *J. Clin. Oncol.*, **10**, 1635–1642.

MILLER, T.P., GROGAN, T.M., DALTON, W.S., SPIER, C.M., SCHEPER, R.J. & SALMON, S.E. (1991). P-glycoprotein expression in malignant lymphoma and reversal of clinical drug resistance with chemotherapy plus high-dose verapamil. *J. Clin. Oncol.*, **9**, 17–24.

NOONAN, K.E., BECK, C., HOLZMAYER, T.A., CHIN, J.E., WUNDER, J.S., ANDRULIS, I.L., GAZDAR, A.F., WILLMAN, C.L., GRIFFITH, B., VON HOFF, D.D. & RONINSON, I.B. (1990). Quantitative analysis of *mdr*1 (multidrug resistance) gene expression in human tumors by polymerase chain reaction. *Proc. Natl Acad. Sci. USA*, **87**, 7160–7164.

PIRKER, R., WALLNER, J., GEISSLER, K., LINKESCH, W., HAAS, O.A., BETTELHEIM, P., HOPFNER, M., SCHERRER, R., VALENT, P. & HAVELOC, L. (1991). MDR1 gene expression and treatment outcome in acute myloid leukaemia. *J. Natl Cancer Inst.*, **83**, 708–712.

RODENBURG, C.J., NOOTER, K., HERWEIJER, H., SEYNAEVE, C., OOSTEROM, R., STOTER, G. & VERWEIJ, J. (1991). Phase II study of combining vinblastine and cyclosporin A to circumvent multidrug resistance in renal cell cancer. *Ann. Oncol.*, **2**, 305–306.

ROTHENBERG, M. & LING, V. (1989). Multidrug resistance: molecular biology and clinical relevance. *J. Natl. Cancer Inst.*, **81**, 907–910.

SONNEVELD, P., DURIE, B.G., LOKHURST, H.M., MARIE, J.P., SOLBU, G., SUCIU, S., ZITTOUN, R., LOWENBERG, G. & NOOTER, K. (1992). Modulation of multi-drug resistant multiple myeloma by cyclosporin. *Lancet*, **340**, 255–259.

SONNEVELD, P. & NOOTER, K. (1990). Reversal of drug resistance by cyclosporin A in a patient with acute myelocytic leukaemia. *Br. J. Haematol.*, **75**, 208–211.

TSURUO, T., OH-HARA, T., IIDA, H., TSUKAGOSHI, S., SATO, Z., MATSUDA, I., IWASAKI, S., OKUDA, S., SHIMIZU, F., SASAGAWA, K., FUKAMI, M., FUKUDA, K. & ARAKAWA, M. (1986). Rhizoxin, a macrocyclic lactone antibiotic, as a new antitumour agent against human and murine tumour cells and their vincristine-resistant sublines. *Cancer Res.*, **46**, 381–385.

UEDA, K., CARDARELI, C., GOTTESMAN, M.M. & PASTAN, I. (1987). Expression of a full-length cDNA for the human *mdr*1 gene confers resistance to colchicine doxorubicin and vinblastine. *Proc. Natl Acad. Sci. USA*, **84**, 3004–3008.

VERELLE, P., MEISSONNIER, F., FONCK, Y., FEILLEL, V., DIONET, C., KWIATKOWSKI, F., PLAGNE, R. & CHASSAGNE, J. (1991) Clinical relevance of immunohistochemical detection of multidrug resistance P-glycoprotein in breast carcinoma. *J. Natl Cancer Inst.*, **83**, 111–116.

WEINSTEIN, R., JAKATE, S.M., DOMINGUEZ, J.M., LEBOVITZ, M.D., KOUKOULIS, G.K., KUSZAK, J.R., KLYSENS, L.F., GROGAN, T.M., SACLARIDES, T.J., RONINSON, I.B. & COON, J.S. (1991). Relationship of the expression of the mdr gene product (P-glycoprotein) in human colon carcinoma to local tumor aggressiveness and lymph node metastasis. *Cancer Res.*, **51**, 2720–2726.

WEINSTEIN, R.S., KUSZAK, J.R., ASHMAN, J.B. & COON, J.S. (1989). P-glycoprotein is targeted to adhesion plaques, refraction fibres and microspikes in drug resistant KB-$V_1$ epidermoid carcinoma cells. *J. Cell Biol.*, **109**, 75a.